# Pregnant human peripheral leukocyte migration during several late pregnancy clinical conditions: a cross-sectional observational study

**DOI:** 10.1186/s12884-016-1204-5

**Published:** 2017-01-10

**Authors:** Jun Takeda, Xin Fang, David M. Olson

**Affiliations:** 1 Departments of Obstetrics and Gynecology, Pediatrics and Physiology, 220 Heritage Medical Research Center, University of Alberta, Edmonton, T6G 2S2 AB Canada; 2 Department of Obstetrics and Gynecology, Juntendo University Faculty of Medicine, Tokyo, Japan

**Keywords:** Chemotactic factor, Leukocyte migration, Preterm birth, Diagnostic

## Abstract

**Background:**

Parturition at term and preterm is characterized by sterile inflammatory processes occurring in the absence of infection whereby peripheral leukocytes infiltrate gestational tissues in response to chemotactic signals. In response to a homing signal, recruited leukocytes undergo diapedesis and extravasate through capillaries, migrating into stromal tissue. There they interact with resident immune and stromal cells to produce a mixture of matrix metalloproteinases, prostaglandins and cytokines including interleukin-1β (IL-1β) and IL-6 that in turn transform the uterus from pregnancy to parturition. Since migration is an early parturitional event our purpose was to study the migration of maternal peripheral blood leukocytes in response to a standard chemotactic signal during several different conditions of late pregnancy.

**Methods:**

We used a cross-sectional observational study design. Subjects were (sTL) spontaneous normal labour delivered vaginally at term, (TNL) elective caesarean section at term without labour, (PTL) preterm in labour, (PTNL) preterm not in labour, (TPTL) threatened preterm labour, and (pPROM) preterm with premature rupture of membranes. Leukocytes (100,000) obtained by venipuncture and chemotactic factor isolated from term labour fetal membranes were placed in the upper and lower halves, respectively, of a Boyden chamber separated by a filter with 3μm pores. Migrated leukocytes were assessed by flow cytometry. The number of leukocytes that migrated in 90 min was the primary outcome measure.

**Results:**

Increased numbers of leukocytes from peripheral blood of women in labour (TL or PTL) or soon to go into labour (PPROM) migrated towards a chemotactic signal than did leukocytes from women not in labour (TNL, PTNL, or TPTL) (*p* < 0.0001). All pPROM delivered within 7d; TPTL delivered >30d. Receiver operating characteristic curve parameters indicated the cut-off point for delivery within 7d to be 37,082 leukocytes with sensitivity 78.1%, specificity 88.9%, positive predictive value 91.4%, negative predictive value 72.7%, and area under the curve 0.83.

**Conclusion:**

Leukocyte migration to a fetal membrane signal varies in a predictable fashion during various clinical situations of late gestation. This principle has the potential to be improved to become a clinical test to predict delivery.

## Background

Parturition at term and preterm is characterized by sterile inflammatory processes occurring in the absence of infection whereby peripheral leukocytes infiltrate gestational tissues in response to chemotactic signals [1–10]. In response to a homing signal, recruited leukocytes undergo diapedesis and extravasate through capillaries into stromal tissue [11]. Then together with resident immune and stromal cells, they produce a mixture of matrix metalloproteinases (MMPs) and cytokines including interleukin-1β (IL-1β) and IL-6 that in turn transform the uterus from pregnancy to parturition by producing prostaglandins, additional MMPs, cytokines, chemokines and uterine transformation proteins [12, 13]. The result is a positive feedback amplification cascade culminating in parturition.

We demonstrated in rats and guinea pigs that the uterus or chorio-amnion produce increasing concentrations of chemotactic factor in late gestation, and there is an increase in leukocyte responsiveness to this factor [14, 15]. Full thickness fetal membranes (amnion, chorion, decidua vera) isolated from spontaneous term labour (TL) patients also is a significant source of chemoattractant of term leukocytes in biological assays [9, 10] and the chemoattractive potency of TL fetal membrane extract is greater than extract obtained from term not in labour patients (TNL). Thus, labour is characterized by two concurrent events: increases in the chemoattractant produced by fetal membrane tissues and, as we demonstrated in rats, an increase in peripheral leukocyte responsiveness (as defined by migration) in response to term chemotactic factor [14]. However, there are no data on whether human peripheral leukocyte migration responsiveness to human TL chemotactic extract increases in late gestation or has characteristics that are consistent with various late pregnancy clinical conditions. Therefore, we hypothesized that human peripheral leukocyte responsiveness to a term chemotactic signal increases as gestation progresses toward the onset of labour at both term and preterm.

## Methods

### Clinical study groups

Pregnant women were recruited from the Royal Alexandra Hospital in Edmonton, Alberta with informed, written consent prior to inclusion in the study. Specific inclusion criteria were used to place women into six study groups for blood sampling: sTL (spontaneous normal labour delivered vaginally at term, 37–42 wk, *n* = 24), TNL (elective caesarean section at term without labour, *n* = 16), PTL (preterm in labour, 22–36 wk, *n* = 10), PTNL (normal women sampled preterm but not in labour, 22–36 wk, *n* = 16), TPTL (threatened preterm labour as identified by uterine contractions or cervical dilation, 22–36 wk, *n* = 11) and pPROM (preterm with premature rupture of membranes but without contractions or dilated cervix, 22–36 wk, *n* = 8). Patients who had disease or medication, multiple pregnancy, fetal congenital disease, and clinical signs of infection (body temperature over 38 °C) were excluded from this study.

### Chemotactic factor isolation

The extracted chemoattractant was used in the migration assay. Fetal membranes were collected from fifteen women who underwent term spontaneous labour without complications. Exclusion criteria were the same as for leukocyte collection. The preparation of the chemoattractant was as published with minor modifications [9, 10, 14, 15]. From each of the fifteen sets of fetal membranes a 4cm square piece was cut. Each of these fifteen square pieces were then washed with 1× phosphate buffered saline (PBS) for 3 times to remove blood and debris, and homogenized with 4mL of Dulbecco’s Modified Eagle’s Medium (DMEM) (Thermo Fischer Scientific Inc., Ottawa, ON, Canada). Peptidase inhibitors were omitted as tests including them demonstrated they had no effect upon the chemoattraction potential of the final preparation. Following centrifugation at 3,000 × *g* for 30 min at 4 °C and then 20,000 × *g* for 2 h at 4 °C (Thermo Scientific™ Sorvall™ ST 16R, Thermo Fischer Scientific Inc., Ottawa, ON, Canada),the supernatants from each piece were collected and pooled together. Protein concentrations (BCA method) were adjusted to 4μg/μL with DMEM. Pooled chemoattractant extracts were aliquoted and stored at−80 °C. For each experiment a vial(s) was placed on ice to thaw. All experiments in this study used vials prepared and frozen from the same batch of chemoattractant and were performed within one year of the original preparation. There was no change in the activity of chemoattractant in that time and the chemoattractant performed similarly to batches prepared at other times (data not shown).

### Blood sampling and leukocyte isolation

Leukocytes were prepared as published with minor modifications [9, 10]. Peripheral blood samples were collected by venipuncture upon recruitment into the study and granting of consent using a standardized protocol for each subject in each of the groups. Leukocytes present in peripheral maternal blood samples drawn into 6mL heparinized tubes were isolated and used in the LMA. Five mL of the anticoagulated blood were combined with 1mL HetaSep (Stemcell, Vancouver, BC, Canada) to remove erythrocytes through sedimentation. Samples were placed in a humidified incubator at 37 °C for 10 min to allow sedimentation of erythrocytes. Approximately 3mL of the top, leukocyte-rich plasma layer were collected and washed with four-fold of 1× PBS. Leukocytes were sedimented using gentle centrifugation (120 × *g* for 10 min at 20 °C without the brake). The supernatant was discarded and leukocytes resuspended in 4mL Hyclone™ Roswell Park Memorial Institute 1640 medium (RPMI) (Thermo Fischer Scientific Inc., Ottawa, ON, Canada) containing 2.0mM L-glutamine. A Bright Line™ hemacytometer (Sigma-Aldrich, St. Louis, MO, USA) was used to count leukocytes. The number of dead leukocytes were recorded using Trypan blue method and the suspension mixture was only used with a viability rate >95%. The leukocyte suspension was diluted using RPMI to a final concentration of 1x10^5^ cells/50μL and used in the LMA within an hour of isolation.

### Leukocyte migration assay (LMA)

**Fig. 1:**
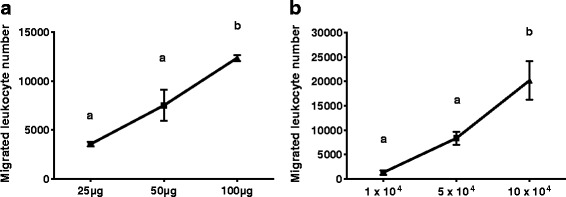
Leukocyte migration assay performance characteristics. **a** Migration as a function of amount of chemoattractant placed in the lower chamber. Increasing amounts of homogenate (determined as total protein in μg) were placed in the lower chamber and migrated (attracted) cells counted after performing the assay as in Methods. Different superscripts denote statistical significance, *p* < 0.05. **b**. Migration as a function of number of leukocytes placed in the upper chamber. Increasing numbers of cells were placed in the upper chamber and migrated (attracted) cells counted after performing the assay as in Methods. Different superscripts denote statistical significance, *p* < 0.05

The procedure used was published [9, 10, 14, 15] with recent modifications to improve the assay performance. Modified Boyden chemotaxis chambers (AP48; Neuro Probe, Gaithersburg, MD, USA) were used in the assay. Twenty-five μL of the chemoattractant extract (100μg total protein) or DMEM as negative control were placed in the lower chamber to create a slightly positive meniscus. A polycarbonate membrane with 3μm pores (Neuro Probe, Gaithersburg, MD, USA) was next placed over the lower chamber followed by a rubber gasket and then the upper chamber. Previously we used a filter with 5μm pores, but we found it allowed too many leukocytes through in the control (blank) tubes resulting in high background counts. We thoroughly tested the system performance with the smaller pores and consequent low blanks (ca. 50–100 cells) and found that the number of cells that migrated was directly dependent upon the amount of chemoattractant placed in the lower chamber and was directly proportional to the number of cells placed in the upper chamber (Fig. 1).

Fifty μL of suspension containing 100,000 leukocytes was pipetted into each well of the upper chamber. The chamber was incubated in a humidified atmosphere at 37 °C containing 5% CO_2_ for 90 min. Following incubation, the upper chamber liquid was removed and the lower chamber samples (into which leukocytes migrated) were individually collected in BD Falcon™ 5mL polystyrene round bottom tubes (Thermo Fischer Scientific Inc., Ottawa, ON, Canada) and combined with 300μL OptiLyse (Beckman Coulter, Mississauga, ON, Canada) to lyse red blood cells and fixed leukocytes. For the subsets analysis, antibodies were added to the tube before adding OptiLyse. Samples were incubated in the dark for 15 min and 1mL 1× PBS was added to each tube. Following centrifugation at 1,000 × *g* for 10 min at 4 °C, the supernatant was discarded and 475μL 1× PBS with 1% formalin was added to fix leukocytes. Lower chamber samples were then kept covered in a 4 °C cold room until quantification using flow cytometry (BD FACSCanto™ II, BD Biosciences, San Jose, CA, USA).

### Flow cytometry

Flow cytometry was used to quantify the number of leukocytes that migrated from the upper chamber down into the lower chamber through the polycarbonate membrane. Each sample had 25μl of CountBright™ Absolute Counting Beads (Life Technologies, Burlington, ON, Canada) added prior to analysis using flow cytometry. The flow cytometer was set to analyze the samples for 30 sec and data were collected using BD FACSDiva software (BD Biosciences, San Jose, CA, USA). Leukocytes were gated based on their forward and side scatter. The total number of leukocytes migrated was calculated using the number of beads added to the tube multiplied by the ratio of leukocytes to beads in the sample aliquot taken by the flow cytometer. The number of leukocytes that migrated in the blank, or negative control, wells containing DMEM was subtracted from all samples in the final calculations.

Leukocyte subtypes that migrated were also analyzed using flow cytometry in a few test cases. Multitest 6-color TBNK Reagent and V450 Mouse Anti-Human CD14 (BD Bioscience, CA, USA) were used for staining cells. Granulocytes were considered as CD45^+^ CD3^−^ CD19^−^ CD16/56^−^ CD14^−^cells.

### Statistics

Data were analyzed and graphs were drawn with GraphPad Prism Version 5.0 software (GraphPad Prism Software, CA, USA). Data were tested for normal distribution and then significance testing was performed with an unpaired *t* test for two-group comparison. One-way ANOVA followed by Tukey’s multiple comparison *post hoc* test was used to detect statistically significant differences between the study groups of over two. *P* < 0.05 was accepted as significant.

## Results

The clinical characteristics of the subjects are described in Table 1. There were no significant differences between the groups of pregnant women with respect to maternal age and parity. Mean gestational age at delivery was significantly higher (*p* < 0.05) in subjects <37 weeks gestation experiencing uterine contractions or cervical ripening (TPTL) or those who were PTNL than when they were tested for leukocyte migration. In each of these groups women delivered more than 30d later. Subjects in the pPROM group all delivered within 7 days of testing leukocyte migration.

**Table 1:** Clinical characteristics of the subjects

	TNL	sTL	PTNL	TPTL	pPROM	PTL
Number	16	24	16	11	8	10
Age	29.0 ± 5.1	28.8 ± 6.4	28.4 ± 5.9	29.3 ± 6.5	26.1 ± 4.4	30.4 ± 4.6
Parity	1.8 ± 1.3	1.5 ± 1.6	1.8 ± 2.0	1.5 ± 1.6	1.9 ± 1.2	1.2 ± 0.9
GA at sampling	39.5 ± 1.1	39.2 ± 1.2	31.9 ± 3.8	29.4 ± 4.0	31.5 ± 2.6	31.8 ± 3.9
GA at delivery			37.1 ± 4.3*	37.5 ± 2.4**	31.7 ± 3.4	

(Mean ± SEM), GA = gestational age (wk)

*p < 0.002, **p < 0.0005 vs. GA at sampling (Unpaired *t* test)

The linearity of leukocyte migration as a function of amount of chemoattractant protein placed in the lower chamber or number of cells placed in the upper chamber is described in Fig. 1. The assay performance was consistent and proportional.

**Fig. 2:**
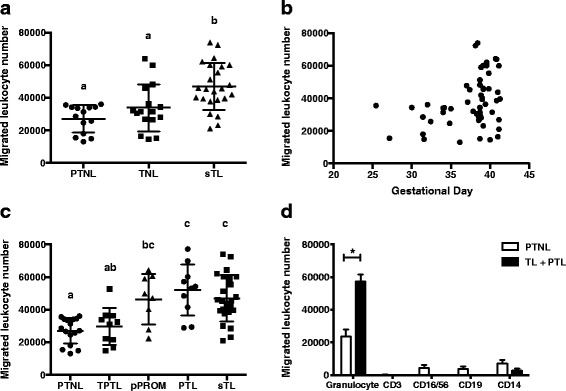
Leukocyte migration in various gestational and clinical groups and leukocyte subtypes migrated. **a**, **b**. Cell migrations from various normal gestational groups, PTNL, TNL and TL (Methods and Table 1). A: cell migrations from individual subjects in groups PTNL vs. TNL vs. sTL. Different superscripts denote statistical difference, *p* < 0.01, one-way ANOVA with Tukey’s post hoc test. B: cell migrations from PTNL, TNL and sTL individual subject values. Pearson’s correlation coefficient on log_10_ transformed data, *r (54)* = 0.306, *p* = 0.025. **c**. Cell migrations from various clinical groups, PTNL vs. TPTL vs. pPROM vs. PTL vs. sTL. Different superscripts denote statistical difference, *p* < 0.0001, one-way ANOVA with Tukey’s post hoc test. **d**. Various leukocyte classes (granulocytes (CD45^+^ CD3^−^ CD19^−^ CD16/56^−^ CD14^−^cells), T-lymphocytes (CD3^−^), NK cells (CD16/56), B-lymphocytes (CD19), monocytes (CD14)) migrated in PTNL (*n* = 4, open bars) and sTL + PTL (*n* = 4, closed bars). The majority were granulocytes. *p < 0.003

Leukocyte responsiveness to term chemotactic signal was analyzed in leukocytes from fifty-four women with normal uncomplicated pregnancies not in labour and <37 weeks gestational age (PTNL), those at term but not in labour (TNL) and those in spontaneous TL (sTL) (Fig. 2a). The data indicated that more sTL leukocytes migrated than PTNL or TNL leukocytes (*p* < 0.01). When the data of Fig. 2a are replotted testing gestational age against leukocyte migration, a sharp upward trend is evident in TNL and sTL women (Pearson’s correlation coefficient on log_10_ transformed data, *r (54)* = 0.306, *p* = 0.025).

We collected leukocytes from healthy subjects in three different not in labour groups at <37 weeks gestation: PTNL, TPTL and pPROM, and compared to women in preterm labour (PTL) and sTL (Fig. 2c). Leukocyte migration was similarly high in the sTL and PTL groups and both were significantly greater (*p* < 0.0001) than the TPTL and PTNL groups. The pPROM group was significantly higher than the PTNL group (*p* < 0.0001), but there was no significant difference between the pPROM and PTL and sTL groups.

Using flow cytometry to analyze the leukocyte subtypes, we determined the highest population of migrated leukocytes was granulocytes in PTNL, PTL, and TL groups and there was a significant difference (*p* < 0.05) in neutrophil migration between PTNL and PTL + TL groups. There was no significant difference found between the groups in terms of subtypes migrated (Fig. 2d). These results informed that assessing the total number of leukocytes migrated provided a reasonable estimate of leukocyte attraction to the standard chemotactic signal.

**Fig. 3:**
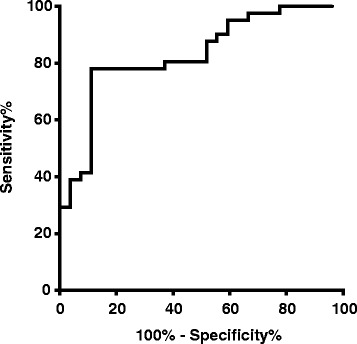
Receiver-operating characteristic curve of leukocyte migration assay

Using all the data from Fig. 2b and delivery within 7 days as the comparator, we examined the parameters of this migration assay by receiver-operating characteristic curve. It identified the cut-off point of delivery within 7 days at 37,082 leukocytes with 78.1% sensitivity, 88.9% specificity, 91.4% positive predictive value, 72.7% negative predictive value, and the area under the curve was 0.83 (Fig. 3).

## Discussion

### Main findings

This is the first report demonstrating that maternal human peripheral leukocytes increase their migration toward a term fetal membrane chemotactic signal when in labour at term or preterm or in the early stages of labour such as pPROM. The exciting aspect of this migration assay is that it has the potential to differentiate those subjects who will not deliver for several weeks from those subjects experiencing symptoms who will deliver within a week.

The percentages of various leukocytes that migrated in the Boyden chamber to the chemotactic signal vary from their distribution when collected and placed into the upper chamber; more neutrophils appear to be more sensitive in labouring women. This suggests that the migrated leukocytes, especially neutrophils, developed greater responsiveness to the chemotactic substance in human fetal membranes than other cells. This would be consistent with reports that demonstrated higher percentages of neutrophils than were in peripheral blood in late gestation or that invaded gestational tissues at term labour [1–3, 16, 17]. However those studies also report increasing invasion of monocytes, suggesting that migration and invasion may be differentially regulated. Thus it is important to note that this assay assesses just one aspect of the more complex process of leukocyte invasion of the uterus prior to delivery.

The increasing responsiveness of the leukocytes to the chemoattractant with labour onset suggests that either there are more leukocyte receptors to the chemoattractant, better coupling of receptors with intracellular signal transduction mechanisms and/or an upregulation of the migration apparatus in the cells. In future investigation we intend to determine whether studying migration of neutrophils only will improve the parameters of the study. At present, though, it is clear that total leukocyte migration is an effective outcome measure for assessing proximity to delivery.

We did not assess the chemical nature of the chemoattractant present in the crude homogenates of human fetal membranes. Data (not shown) indicates it is also present in uterine tissues from mice and rats and these rodent chemoattractants are able to attract human leukocytes. We are in the process of identifying it chemically with expert collaborators. Preliminary analysis suggests it is not one of the common chemokines and isolating it has proven difficult. We will continue our analysis using a variety of biochemical isolation techniques. Considerable more work is required before it will be identified.

The receiver-operating characteristic curve utilized all the data available (Fig. 3) to determine preliminary parameters of the assay. While preliminary, these data are highly encouraging in that the positive predictive value, negative predictive value, sensitivity and specificity are very high. They suggest that with further improvement in terms of simplifying the technical aspects so it can become a point of care test, the assay described here may form the basis for a future clinical diagnostic test.

### Strengths and limitations

We demonstrated an interesting and potentially clinically applicable characteristic of circulating leukocytes in late gestation women and those in various clinical situations. Further refinement and improvement will both reveal basic biological mechanisms of late gestation in normal and inflammatory situations plus possibly lead to an informative diagnostic test to predict preterm birth risk. One limitation of this study is that it is only cross-sectional with separate subjects in each determination. Future work comprising longitudinal samples from a single subject during pregnancy, at term or preterm, threatened preterm or ruptured membranes would be very informative.

### Interpretation

Leukocyte migration has the potential with further development, refinement and simplification to become a diagnostic test that would be valuable for identifying asymptomatic women who may be at risk for preterm birth. In addition, many symptomatic women present with signs of preterm labor but only 10–20% will deliver preterm. It is important from a treatment and also a health care costing perspective to know which of these women to treat or admit to hospital, and which to send home. Existing tests, the fetal fibronectin (fFN) test and the insulin-like growth factor binding protein-1 (IGFBP-1) test, have high negative prediction values, but low positive predictive values [17–19]. They indicate who will not deliver, but they do not indicate who will deliver within one to two weeks. A test with a high positive predictive value, along with a reasonable negative predictive value, would be a significant advance in the management of pregnant women.

## Conclusion

Leukocyte migration to a fetal membrane signal varies in a predictable fashion during various clinical situations of late gestation. This principle has the potential to be improved to become a clinical test to predict delivery.

## Abbreviations

BC: British Columbia
BCA: Bicinchoninic acid assay
C: Celsius
Ca: Approximately
CA: California
CIHR: Canadian Institutes of Health Research
Cm: Centimeter
D: Day
DMEM: Dulbecco’s minimum essential medium
Fig.: Figure
G: Gram
GA: Gestational age (in weeks)
GAPPS: Global Alliance for the Prevention of Prematurity and Stillbirth
H: Hour
IL-1β: Interleukin 1β
IL-6: Interleukin 6
LMA: Leukocyte migration assay
MMP: Matrix metalloproteinase
Mg: Microgram
μL: Microliter
μm: Micrometer
mL: Milliliter
min: minute
MO: Missouri
ON: Ontario
P: Probability
PBS: Phosphate buffered saline
PCT: Patent Cooperation Treaty
pPROM: Preterm premature rupture of membranes
PTL: Preterm labor
PTNL: Preterm not in labor
RPMI: Roswell Park Memorial Institute
sTL: Spontaneous term labor
TNL: Term not in labor
TPTL: Threatened preterm labor
USA: United States of America
Wk: Week

## References

[1] Bokstrom H, Brannstrom M, Alexandersson M, Norstrom A (1997). Leukocyte subpopulations in the human uterine cervical stroma at early and term pregnancy. Hum Reprod.

[2] Osman I, Young A, Ledingham MA, Thomson AJ, Jordan F, Greer IA, Norman JE (2003). Leukocyte density and pro-inflammatory cytokine expression in human fetal membranes, decidua, cervix and myometrium before and during labour at term. Mol Hum Reprod.

[3] Thomson AJ, Telfer JF, Young A, Campbell S, Stewart CJ, Cameron IT, Greer IA, Norman JE (1999). Leukocytes infiltrate the myometrium during human parturition: further evidence that labour is an inflammatory process. Hum Reprod.

[4] Ledingham MA, Thomson AJ, Jordan F, Young A, Crawford M, Norman JE (2001). Cell adhesion molecule expression in the cervix and myometrium during pregnancy and parturition. Obstet Gynecol.

[5] Romero R, Espinoza J, Goncalves LF, Kusanovic JP, Friel LA, Nien JK (2006). Inflammation in preterm and term labour and delivery. Semin Fetal Neonatal Med.

[6] Winkler M, Kemp B, Fischer DC, Ruck P, Rath W (2003). Expression of adhesion molecules in the lower uterine segment during term and preterm parturition. Microsc Res Tech.

[7] Bollopragada S, Youssef R, Jordan F, Greer I, Norman J, Nelson S (2009). Term labor is associated with a core inflammatory response in human fetal membranes, myometrium, and cervix. Am J Obstet Gynecol.

[8] Young A, Thomson AJ, Ledingham M, Jordan F, Greer IA, Norman JE (2002). Immunolocalization of proinflammatory cytokines in myometrium, cervix, and fetal membranes during human parturition at term. Biol Reprod.

[9] Gomez-Lopez N, Estrada-Gutierrez G, Jiminez-Zamudio L, Vega-Sanchez R, Vadillo-Ortega F (2009). Fetal membranes exhibit selective leukocyte chemotaxic activity during human labor. J Reprod Immunol.

[10] Gomez-Lopez N, Vadillo-Perez L, Nessim S, Olson DM, Vadillo-Ortega F (2011). Choriodecidua and amnion exhibit selective chemotaxis during term labor. Am J Obstet GynecolAmerican.

[11] Chang JR, Tees DFJ, Hammer DA (2000). The state diagram for cell adhesion under flow: leukocyte rolling and firm adhesion. Proc Natl Acad Sci.

[12] Challis JR, Lockwood CJ, Myatt L, Norman JE, Strauss JF, Petraglia F (2009). Inflammation and pregnancy. Reprod Sci.

[13] Myatt L, Sun K (2010). Role of fetal membranes in signalling of fetal maturation and parturition. Int J Dev Biol.

[14] Gomez-Lopez N, Tanaka S, Zaeem Z, Metz GA, Olson DM (2013). Maternal circulating leukocytes display early chemotactic responsiveness during late gestation. BMC Pregnancy Childbirth.

[15] Gomez-Lopez N, Tong WCH, Arenas-Hernandez M, Tanaka S, Hajar O, Olson DM, Taggart MJ, Mitchell B (2015). Chemotactic activity of gestational tissues through late pregnancy, term labor and RU486-induced preterm labor in guinea pigs. Am J Reprod Immunol.

[16] Yuan M, Jordan F, McInnes IB, Harnett MM, Norman JE (2009). Leukocytes are primed in peripheral blood for activation during term and preterm labour. Mol Hum Reprod.

[17] Gomez-Lopez N, StLouis D, Lehr MA, Sanchez-Rodriguez EN (2014). Immune cells in term and preterm labor. Cell Mol Immunol.

[18] Cooper S, Lange I, Wood S, Tang S, Miller L, Ross S (2012). Diagnostic accuracy of rapid phIGFBP-I assay for predicting preterm labor in symptomatic patients. J Perinatol.

[19] Sananes N, Langer B, Gaudineau A, Kutnahorsky R, Aissi G, Fritz G, Boudier E, Viville B, Nisand I, Favre R (2014). Prediction of spontaneous preterm delivery in singleton pregnancies: Where are we and where are we going? A review of literature. J Obstet Gynaecol.

